# Peritoneal adipose stem cell-derived extracellular vesicles mediate the regulation of ovarian cancer cell proliferation and migration through EGFR-NF-κB signaling

**DOI:** 10.1016/j.gendis.2024.101283

**Published:** 2024-03-28

**Authors:** Lian Wang, Ning Luo, Jihui Zhu, Zubaidan Sulaiman, Wenhan Yang, Ke Hu, Guihai Ai, Weihong Yang, Xiaowen Shao, Shengkai Jin, Xue Zhang, Yantao Fan, Dan Deng, Zhongping Cheng, Zhengliang Gao

**Affiliations:** a Department of Gynecology and Obstetrics, Gynecologic Minimally Invasive Surgery Research Center, Shanghai Tenth People's Hospital, Tongji University School of Medicine, Shanghai 200072, China; b Department of Anesthesiology, Shanghai Gongli Hospital, Naval Military Medical University, Shanghai 200135, China; c Shanghai Engineering Research Center of Organ Repair, Shanghai University School of Medicine, Shanghai 200444, China; d Institute of Geriatrics (Shanghai University), Affiliated Nantong Hospital of Shanghai University (The Sixth People's Hospital of Nantong), Shanghai University School of Medicine, Nantong, Jiangsu 216002, China; e Department of Dermatology, Shanghai Children's Medical Center, School of Medicine, Shanghai Jiao Tong University, Shanghai 200127, China; f Department of Dermatology, Xinhua Hospital, Shanghai Jiaotong University School of Medicine, Shanghai 200092, China; g China-Japan Friendship Medical Research Institute, School of Medicine, Shanghai University, Shanghai 200444, China

**Keywords:** Adipose-derived stem cells, EGFR/NK-κB axis, Extracellular vesicles, Metastasis, Ovarian cancer

## Abstract

Peritoneal dissemination frequently develops in patients with ovarian cancer (OC) and is associated with recurrence and metastasis. However, the cellular components and mechanisms supporting OC peritoneal metastasis are poorly understood. To elucidate these, we utilized RNA sequencing to investigate the cellular composition and function. Insights from transcriptome analyses suggested that OC cells from malignant ascites persisted in a quiescent state of low metabolic activity and after metastases to the peritoneum, arrested OC cells were reactivated and induced back to the cell cycle, suggesting that the peritoneum served as a favor tumor microenvironment. To elucidate the mechanisms, we then developed long-range migration and competitive inhibition assays and showed that peritoneal adipose-derived stem cells-derived extracellular vesicles (ADSCs-EVs) mediated preferential migration of OC cells toward peritoneal ADSCs but not other representative cells from the peritoneal cavity. In line with phenotypic changes, transcriptomic analysis revealed that patient peritoneal ADSCs-EVs stimulated the expression of numerous genes associated with OC cell proliferation and migration; among them, the epidermal growth factor receptor (EGFR) and nuclear factor kappa B (NF-κB) signaling pathways were highly enriched. We also found that peritoneal ADSCs produced and secreted key EGFR signaling molecules, including EGF and EGFR, into ADSCs-EVs. Upon fusion with OC cells, ADSCs-EVs up-regulated the EGFR-NF-κB axis and promoted OC cell proliferation and migration. Interference with either ADSCs-EVs production or EGFR signaling abolished the proliferation and migration effect. The results show that ADSCs modulate OC cell proliferation and migration at multiple layers, constituting a key mechanism in OC progression.

## Introduction

Ovarian cancer (OC), the most lethal gynecological malignancy, is the fifth leading cause of cancer-related death in women.^1^^,^^2^ More than 80% of patients are diagnosed at advanced stages (stages III and IV according to the International Federation of Gynecology and Obstetrics system) with a 5-year survival rate of less than 30%, which has remained essentially unimproved since 1980.^2^ The main challenges of ovarian cancer combat are relapse and metastasis, which quickly develop after debulking surgery and standard chemotherapy.^3^^,^^4^ Hence, it is both crucial and urgent to mechanistically investigate relapse and metastasis and foster novel therapeutic strategies.

Before and during colonization, tumor cells communicate with and reprogram cells at potential distant metastatic sites, and these interactions in turn provide feedback to modulate tumor cell behaviors.^5^ Malignant ascites, a hallmark of advanced OCs, develop in one-third of patients and contain various cellular and acellular components associated with poor prognosis, providing a nurturing and bridging environment for cancer progression and peritoneal dissemination.^6^ Both primary and relapsed OCs show a strong tropism for adipose tissues, and approximately 80% of OCs metastasize to the omentum, essentially a fat pad.^7^, ^8^, ^9^, ^10^ Hence, it has been proposed that adipocytes play important roles in OC metastasis,^11^ facilitating exogenous fatty acid uptake into OC cells via up-regulation of fatty acid receptor expression and/or by directly serving as an energy resource for the rapid progression of cancer.^12^^,^^13^ Adipose-derived stem cells (ADSCs) are the second major component of adipose tissues, which normally act as both resident stem cells and supporting stromal cells in tissues critical for regeneration and homeostasis.^14^ ADSCs are capable of secreting numerous factors with diverse biological effects, including leptin,^15^^,^^16^ matrix metalloproteinases,^17^^,^^18^ cytokines,^19^^,^^20^ and chemokines,^21^ manifesting strong growth stimulatory, immunomodulatory, and angiogenic effects. Thus, ADSCs may promote cancer progression and metastasis, as emerging studies indicate.^14^^,^^22^

In the present study, we thoroughly investigated the potential involvement of ADSCs in OC progression and metastasis. Transcriptomic profiling of primary OC tissues, malignant ascites, and peritoneal OC tissues provided strong evidence that OC cells disseminated to the peritoneum were reactivated and stimulated after being forced into a quiescent state with low metabolic activity by malignant ascites. To validate this hypothesis, we employed colony formation assays, transwell migration assays, and wound healing assays, confirming that peritoneal ADSCs indeed enhance OC cell proliferation and migration capabilities. Subsequently, we further developed and performed long-range migration and competitive inhibition assays, revealing that peritoneal ADSCs promoted OC cell proliferation and migration through extracellular vesicles (EVs)-mediated long-range regulation. Through transcriptome analysis, we found that treatment with ADSCs-derived extracellular vesicles (ADSCs-EVs) led to widespread alterations in gene expression related to OC cell proliferative and migratory properties, including several key players in epidermal growth factor receptor (EGFR) and nuclear factor kappa B (NF-κB) signaling. This observation prompted us to further examine and uncover the capacity of ADSCs to actively secrete and deliver key EGFR signaling molecules including epidermal growth factor (EGF) and EGFR to OC cells at a distance by means of ADSCs-EVs. Upon arrival and fusion with OC cells, ADSCs-EVs up-regulate a range of tumorigenic pathways including the EGFR-NF-κB axis, thereby globally reprogramming OC cells and promoting their proliferation and migration. We then demonstrated that inhibiting ADSCs-EVs production by using small molecule inhibitor GW4869, as well as knocking down EGFR expression with shRNAs, could both effectively prevent the proliferation and migration effects induced by ADSCs-EVs. Together our results suggest that peritoneal ADSCs modulate OC progression and metastasis through ADSCs-EVs-mediated long-range regulation of EGFR and NF-κB signalling pathways.

## Materials and methods

### Collection of patient OC and ascites samples

The collection of ovarian cancer tissues and ascites was approved by the Ethics Committee of Shanghai Tenth People's Hospital. In total, we collected 22 tumor samples from 9 patients with ovarian cancer, which comprised 9 primary OC tissues, 4 samples of malignant ascites, and 9 peritoneal OC tissues (as summarized in Table 1).

**Table 1:** Information of ovarian cancer tissue sample.

Patients	Ovarian cancer	FIGO stage	Sample
A76302	HGSC	IIIc	Primary ovarian cancer, peritoneal ovarian cancer, ascites
B06435	HGSC	IIIc	Primary ovarian cancer, peritoneal ovarian cancer, ascites
B41519	HGSC	IIIc	Primary ovarian cancer, peritoneal ovarian cancer, ascites
B42181	HGSC	IIIc	Primary ovarian cancer, peritoneal ovarian cancer, ascites
B48144	HGSC	IIIc	Primary ovarian cancer, peritoneal ovarian cancer
A5006982	LGSC	Ia	Primary ovarian cancer, peritoneal ovarian cancer
A97477	HGSC	IIIc	Primary ovarian cancer, peritoneal ovarian cancer
B28534	HGSC	IIIc	Primary ovarian cancer, peritoneal ovarian cancer
A99509	HGSC	IIIc	Primary ovarian cancer, peritoneal ovarian cancer

**Notes:** FIGO, the International Federation of Gynecology and Obstetrics; LGSC, low-grade serous carcinoma; HGSC, high-grade serous carcinoma.

### Isolation of ADSCs and adipocytes

Isolation and identification of ADSCs were performed as previously reported.^23^ Briefly, adipose tissues were manually minced and digested with 1 mg/mL collagenase I solution (Sigma, C0130) at 37 °C for 1 h. The dissociated adipose tissues were filtered through a 100-μm cell strainer (BioFil, CSS-013-100) to remove undigested tissue and were then centrifuged at 2000 rpm for 5 min to obtain a stromal vascular fraction containing ADSCs.^24^ ADSCs were cultured in DMEM/F12-10% fetal bovine serum (Gibco, 10099-141) and subcultured at 90% confluence every 3 days. Flow cytometry (Beckman Coulter, CytoFLEX LX) analysis and trilineage differentiation assays were performed to confirm the identity of ADSCs.^25^

During the ADSC isolation process, adipocytes, visible as a white ring layer in the upper phase of the supernatant, were carefully transferred to a new centrifuge tube, washed with DMEM/F12–0.1% bovine serum albumin, and centrifuged at 100 *g* for 5 min. Adipocytes were carefully collected, seeded, and cocultured with OC cells for further transwell assays.

### Human ovarian cancer and control cell culture

The human OC cell line SKOV3 was cultured and maintained in RPMI 1640 medium supplemented with 10% fetal bovine serum at 37 °C in 5% CO_2_. Normal ovarian surface epithelial cell line IOSE80 and human embryonic kidney cell line 293T cells were cultured and maintained in DMEM supplemented with 10% fetal bovine serum at 37 °C in 5% CO_2_.

### Isolation of extracellular vesicles

Isolation of extracellular vesicles (EVs) was achieved by the Lässer protocol with modifications.^26^ Briefly, ADSCs were washed with phosphate-buffered saline solution (PBS) and cultured in DMEM/F12 supplemented with EVs-depleted fetal bovine serum for 48 h. The medium was then collected and centrifuged at 300 *g* at 4 °C for 10 min to remove detached ADSCs. The supernatants were transferred and centrifuged at 10,000 *g* for 30 min to remove cell debris and organelles and then at 100,000 *g* for 120 min to pellet EVs. After resuspension, the EV pellets were washed with ice-cold PBS and ultracentrifuged again at 100,000 *g* for 120 min. Finally, the EV pellets were resuspended in 500 μL PBS and used for further analyses.

### Nanoparticle tracking analysis

The particle size and concentration of isolated EVs were determined with a ZetaView PMX 110 instrument (Particle Metrix, Meerbusch, Germany) and analyzed with ZetaView 8.04.02 software. I. Polystyrene particles of 110 nm were used to calibrate the ZetaView system at 25 °C. Nanoparticle tracking analysis measurements were taken and performed at 11 positions.

### Transmission electron microscopy

Aliquots of 20 μL of EVs were dropped onto formvar-carbon coated electron microscopy grids (200 mesh Cu), fixed overnight with 4% paraformaldehyde, and negatively stained with 2% uranyl acetate aqueous solution for 10 min. Images were acquired with a Tecnai G2 Spirit BioTWIN transmission electron microscope.

### PKH67 labeling and uptake of EVs

A PKH67 Green Fluorescent Cell Linker Kit (Sigma, PKH67GL) was used to label ADSCs-EVs following the manufacturer's instructions. Briefly, 50 μL of ADSCs-EVs was diluted with 150 μL of DMEM/F12 and then mixed with 20 μL of PKH67 staining solution. After a 15-min incubation at 37 °C, 15 mL of DMEM/F12 was added to terminate the staining reaction. Then, the mixture was transferred to an Amicon® Ultra-15 100 kDa centrifugal filter (Merck Millipore, UFC910024) and centrifuged at 4000 *g* and 4 °C for 30 min to remove excess dye and harvest the labeled ADSCs-EVs.^27^

For the uptake assay, 20 μL labeled ADSCs-EVs per well were added to OC cells in a 24-well plate and incubated at 37 °C for 2 h. Then, the OC cells were washed with PBS and fixed with 4% paraformaldehyde. The endoplasmic reticulum was stained with an ER-Tracker Red Kit (Beyotime, C1041),^28^ and nuclei were stained with 4′,6-diamidino-2-phenylindole. The staining was visualized and analyzed with an Olympus BX53 microscope.

### Transwell coculture assays

Transwell co-culture assays were employed to evaluate the effects of adipocytes, stromal vascular fraction, ADSCs, ADSCs-conditioned medium (ADSCs-CM), ADSCs-EVs, and EGF on the proliferation and migration of OC cells. Briefly, 2 × 10^4^ adipocytes, stromal vascular fraction, ADSCs, or appropriate volumes of ADSCs-CM, ADSCs-EVs, and EGF were added to the lower chambers of the transwell inserts (pore size: 8 μm for migration assay and 0.4 μm for proliferation assay), and 1 × 10^4^ OC cells were seeded in the upper chambers. After a 48-h incubation, OC cells in the upper chambers were fixed with 4% paraformaldehyde, stained with 0.5% crystal violet, and counted to assess proliferation. OC cells in the lower chambers that migrated through the membrane were stained and counted to assess migration.

### Long-range migration and competition assays

To determine whether ADSCs remotely regulate the migration of OC cells, we developed a long-range migration assay. Aliquots of 2 × 10^4^ ADSCs and OC cells in 20 μL RPMI 1640 medium were seeded as isolated dots separated from each other in the same wells in a 24-well plate. The cells were allowed to adhere during a 1-h incubation at 37 °C in 5% CO_2_, and 500 μL medium per well was then carefully added without disturbing the adherent cells. After another 24-h incubation, the cells were fixed with 4% paraformaldehyde and stained with 0.5% crystal violet. Mutual migration was analyzed under an Olympus BX53 microscope. For competition assays, equal numbers of competitive cells (2 × 10^4^ cells for ADSCs, IOSE cells, and 293T cells) were seeded at the same distance from OC cells at the centers of equilateral triangles.

### Wound healing assay

A wound healing assay was used to examine OC cell migration under different conditions. Briefly, scratch wounds were created when OC cells reached 80% confluence. Cell debris was removed by PBS washes, and the cells were switched to the indicated experimental conditions. At 0 and 24 h after wounding, images were acquired and evaluated for wound healing by microscopy using a 4× objective.

### Western blotting analysis

Western blotting analysis was performed as previously described.^23^ Briefly, total protein extraction was prepared in RIPA lysis buffer with concentrations determined by a BCA protein assay kit (Bio-Rad, 500-0201). Aliquots of 20 μg proteins per sample were separated by 10% SDS-PAGE, transferred to Immobilon-P PVDF membranes (Merck Millipore, IPVH00010), and blocked with 3% bovine serum albumin in PBST (0.3% Tween 20 in PBS). Primary antibody incubations were performed at 4 °C overnight (Table 2; anti-GAPDH: 1:3000; anti-EGFR, anti-NF-κB, anti-RELA, and anti-TAK1: 1:1000) followed by a 2-h secondary antibody incubation (HRP-conjugated anti-rabbit IgG: 1:3000) at room temperature. Enhanced chemiluminescence substrate was used to visualize immunoreactions in an ImageQuant LAS 4000 system.

**Table 2:** Antibody resource table.

Antibodies	Source	Identifier
GAPDH rabbit pAb	Abclonal	AC001
EGFR rabbit pAb	Abclonal	A11351
NF-κB1 rabbit pAb	Abclonal	A6667
RELA rabbit mAb	Abclonal	A19653
TAK1 rabbit pAb	Abclonal	A12022
HRP-linked anti-rabbit IgG antibody	CST	7074S

### Immunofluorescence staining

OC cells were seeded on coverslips in 24-well plates, and after desired treatments, they were fixed with 4% paraformaldehyde for 15 min, permeabilized in 0.3% Triton X-100 for 15 min, and blocked with 3% bovine serum albumin at room temperature for 1 h. Primary antibody incubations were performed at 4 °C overnight (Table 3; anti-Ki67: 1:1000; anti-EGFR, anti-NF-κB, anti-CXCR4, anti-CXCR2, anti-CCR1, and anti-CCR7: 1:300) followed by secondary antibody staining (1:1000) at room temperature for 1 h. Nuclei were counterstained with 4′,6-diamidino-2-phenylindole. Images were acquired using an Olympus BX53 microscope and analyzed using NIS-Elements Viewer 4.5 software.

**Table 3:** Antibody resource table.

Antibodies	Source	Identifier
Ki-67 rabbit mAb	ThermoFisher Scientific	MA5-14520
EGFR rabbit pAb	Abclonal	A11351
NF-κB1 rabbit pAb	Abclonal	A6667
CXCR2 rabbit pAb	Abclonal	A3301
CXCR4 rabbit pAb	Abclonal	A12534
CCR1 rabbit pAb	Abclonal	A18341
CCCR7 rabbit pAb	Abclonal	A0121
Cy™3 donkey anti-rabbit IgG (H + L)	Jackson ImmunoResearch	711-165-152

### EGFR overexpression and shRNA knockdown

EGFR overexpression (pLenti-CMV-EGFR(WT)-GFP-Puro), shRNA1 (GCCAAGCCAAATGGCATCTTT), shRNA2 (GCTGAGAATGTGGAATACCTA), and shRNA3 (GCCACAAAGCAGTGAATTTAT) plasmids were purchased from Public Protein/Plasmid Library Company (Jiangsu, China) and verified by sequencing. EGFR knockdown and overexpression were achieved by lentiviral transduction of OC cells followed by puromycin selection. Successful knockdown and/or overexpression were verified by immunofluorescence staining, quantitative reverse transcription PCR (qRT-PCR), and western blot analysis.

### Enzyme-linked immunosorbent assay

The concentrations of EGF (RK00024HS; ABclonal, China) in ADSCs-CM and ADSCs-EVs were measured by enzyme-linked immunosorbent assay according to the manufacturer's instructions.

### qRT-PCR

Total RNA samples were prepared using TRIzol reagent (15596026; Invitrogen, Carlsbad, CA, USA). cDNA synthesis was accomplished with a FastQuant RT Kit (KR106; Tiangen, Beijing, China), and qRT-PCR was performed with SYBR Green Master Mix (FP209; Tiangen, China) using a 7500 Real-Time PCR System. The 18S gene was included as the internal control. The primers used are listed in Table 4.

**Table 4:** Primers used for real-time PCR.

Gene	Forward primer	Reverse primer
ALOX5	TGGCGCGGTGGATTCATAC	CCAGTCGTCATTCAGCCAGT
COX2	CTGCGCCTTTTCAAGGATGG	CCCCACAGCAAACCGTAGAT
CCR1	CCGACTGCCATCTTGGACTT	TCCTCCCAACCCCCTATCAG
CCR7	ACAGCCTTCCTGTGTGGTTTTA	AATGACAAGGAGAGCCACCAC
CXCR2	ACATTCAGAGACAGAAGGTGG	TGAGTGCTGGGGATTTTCACA
CXCR4	GCAGCAGGTAGCAAAGTGAC	GCCCATTTCCTCGGTGTAGT
EGFR	TTGCCGCAAAGTGTGTAACG	GAGATCGCCACTGATGGAGG
NF-κB1	TGAGGTCTCTGGGGGTACAG	GCCTGAGAGGTGGTCTTCAC
TAK-1	AACCACAGGCAACGGACAG	ACACTGGGACTGGATGACCT
RELA	TCCAGTGTGTGAAGAAGCGG	TCCCCACGCTGCTCTTCTAT
IKKB	CTTCCCTGACAACGCAGACA	TGGCAATCTGCTCACCTGTT
CCNE1	CCCGCAGGCAGTCCACTTCA	CGGCTCGCTCCAGGAA
CCNB2	GGCTGGTACAAGTCCACTCC	CTTCTTCCGGGAAACTGGCT
CDK2	TGGATGCCTCTGCTCTCACTG	GAGGACCCGATGAGAATGGC
PKM	TACCATTGTACCATGCGGAGA	CTTGATGAGCCCAGTTCGGA
LDHB	TCTCTCCTGTCTCTGGCTGAT	CTCTCCCCTTCTTGCTGACG
CXCL2	TTCACAGTGTGTGGTCAACAT	TCTCTGCTCTAACACAGAGGGA
CXCL3	TGAATGTAAGGTCCCCCGGA	CACCCTGCAGGAAGTGTCAA
18S	GCCGCTAGAGGTGAAATTCTTG	CATTCTTGGCAAATGCTTTCG

### RNA sequencing

Total RNA samples were extracted from primary OC tissues, peritoneal OC tissues, ascites tissues, ADSCs-EVs treated SKOV3 cells, and control SKOV3 cells using TRIzol reagent (Invitrogen, catalog no. 15596018), and the RNA integrity number was determined with a Bioanalyzer 2100 and an RNA 6000 Nano LabChip Kit (Agilent, CA, USA, 5067-1511). mRNA was enriched from total RNA samples with an RNA integrity number greater than 8 using oligo (dT) magnetic beads prior to cDNA synthesis, library construction, and paired-end RNA sequencing on an Illumina NovaSeq 6000. For DESeq2 analysis, after mapping the reads to the human genome using HISAT2^29^ and quantifying gene expression levels with FeatureCounts,^30^ raw read counts were used as input for DESeq2. We utilized the default normalization method provided by DESeq2 to correct for potential differences in library sizes that could introduce compositional biases in the RNA-sequencing data. Applying the criteria of |log2FC| >1 and *P*_adj_ < 0.05, we successfully identified differentially expressed genes between primary OC tissue, peritoneal OC tissue, and ascites, and differentially expressed genes of ADSCs-EVs treated SKOV3 cells and untreated control SKOV3 cells. Pathway enrichment analysis was performed using the g:Profiler web tool.^31^ The STRING database was used to study protein–protein interactions.^32^, ^33^, ^34^ Gene set enrichment analysis was performed using GSEA 4.1.0 software.^2^^,^^35^

### Statistical analysis

All data are reported as the mean ± standard error of the mean unless otherwise specified. GraphPad Prism version 5.01 was used for the analysis of variance. Quantitative data were analyzed using a two-tailed unpaired Student's *t*-test. Statistical significance was set at ^∗^*P* < 0.05, ^∗∗^*P* < 0.01, ^∗∗∗^*P* < 0.001, and ^∗∗∗∗^*P* < 0.0001.

## Results

### Potential reactivation and stimulation of disseminated OC cell metabolism and proliferation by peritoneal adipose tissue

**Figure 1:**
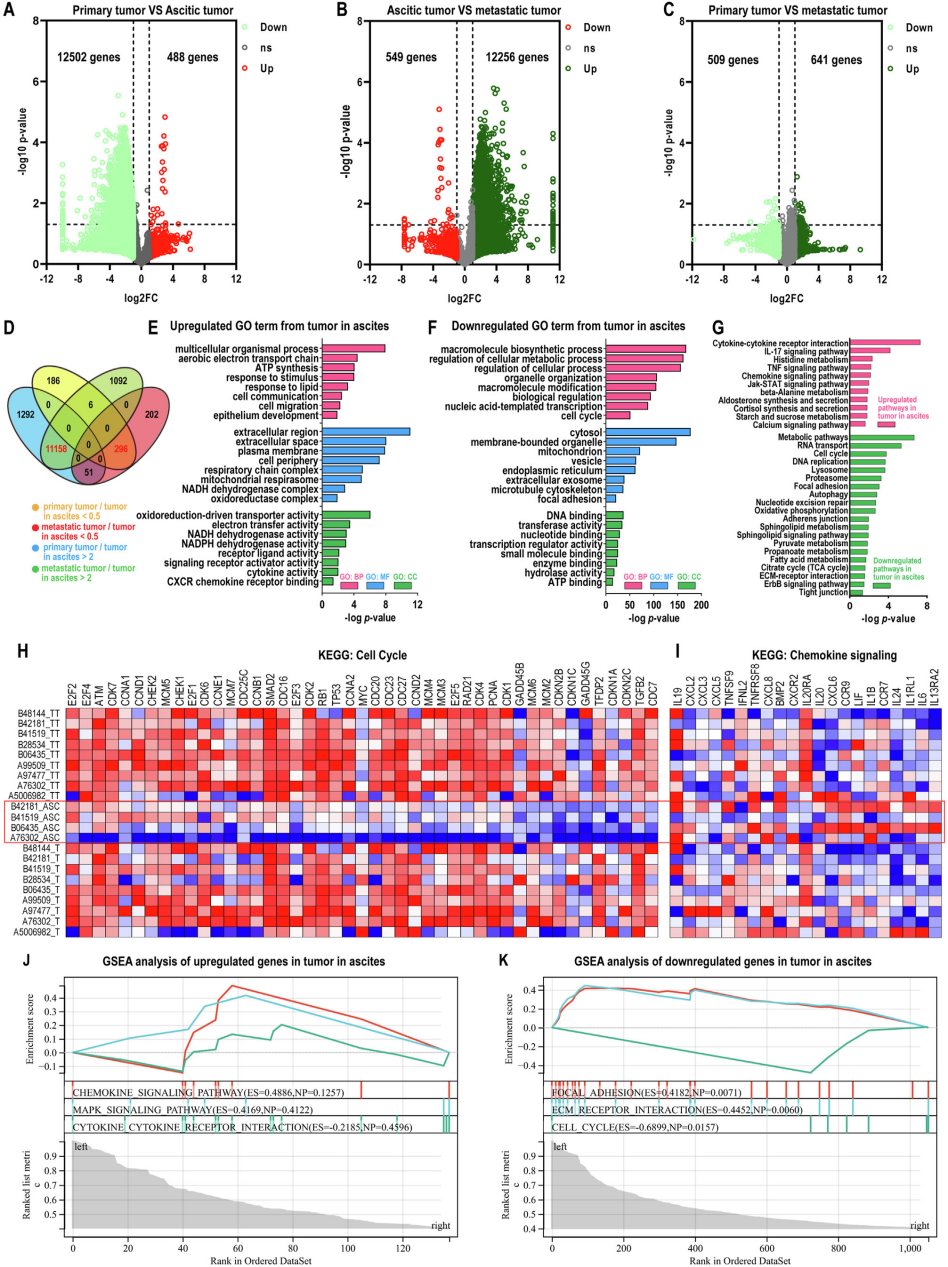
Transcriptomic profiling suggests potential reactivation and stimulation of disseminated ovarian cancer (OC) cell metabolism and proliferation by peritoneal adipose tissue. **(A**–**C)** The volcano plot showing differentially expressed genes between primary and malignant ascites OC tissues (A), malignant ascites and peritoneal OC tissues (B), and primary and peritoneal OC tissues (C). **(D)** The Venn diagram showing differentially expressed genes of pairwise comparisons. Yellow, genes up-regulated in malignant ascites compared with primary OC tissues; blue, genes down-regulated in malignant ascites compared with primary OC tissues; red, genes up-regulated in malignant ascites compared with peritoneal OC tissues; green, genes down-regulated in malignant ascites compared with peritoneal OC tissues. The number of differentially expressed genes is indicated in the diagram. **(E, F)** GO term analysis of the 259 overlapping genes up-regulated (E) and the 11,158 overlapping genes down-regulated in malignant ascites (F). **(G)** KEGG pathway analysis of the 259 up-regulated genes and the 11,158 down-regulated genes. **(H, I)** The heatmap showing genes related to the cell cycle (H) and chemokine signaling (I). **(J, K)** GSEA showed significant enrichment of up-regulated genes related to the chemokine signaling pathway, MAPK signaling pathway, and cytokine–cytokine receptor interaction (J) and down-regulated genes associated with focal adhesion, extracellular matrix (ECM) receptor interaction, and cell cycle in malignant ascites (K).

**Figure S1:**
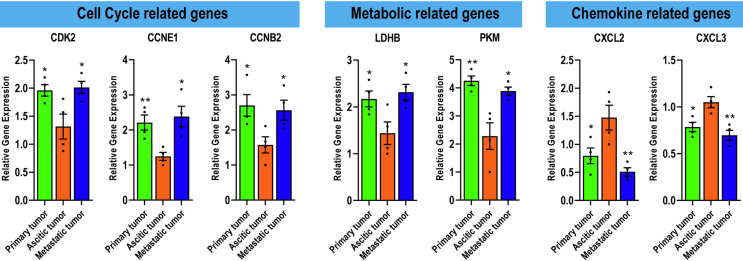
The validation of representative gene expression of RNA-sequencing data by quantitative reverse transcription PCR.

Ovarian cancer (OC) typically metastasizes via ascites implantation and most commonly disseminates to the omentum. As OC cells transition across these distinct microenvironments, from ovarian tissue to ascites and then to peritoneal adipose tissue, their cellular states and inherent properties might undergo substantial changes. To validate this hypothesis, we performed transcriptomic profiling on OC tissues from three different environments. In total, we sequenced 22 samples, including 9 primary OC tissues, 4 malignant ascites, and 9 peritoneal OC tissues derived from 9 patients. Compared with primary tumor, malignant ascites down-regulated 12,502 and up-regulated 488 genes (Fig. 1A). As OC cells disseminated to the omentum from malignant ascites, 12,256 genes were up-regulated and 549 genes were down-regulated (Fig. 1B). As a result, the expression of most of the differentially expressed genes between primary tumors and malignant ascites was reversed to a primary tumor-like level in the omentum (Fig. 1C). Closer examination showed that 11,158 genes were down-regulated and 259 genes were up-regulated in ascitic OC cells compared with both primary and peritoneal OC cells (Fig. 1D). Gene Ontology (GO) analysis revealed that macromolecular biosynthesis, cellular metabolism, and cell cycle activity were significantly repressed during OC metastasis to ascites and restored as OC disseminated to the omentum (Fig. 1F). The Kyoto Encyclopedia of Genes and Genomes (KEGG) pathway analysis further confirmed the down-regulation of metabolism, cell cycle, DNA replication activities, and associated ErbB signalling pathway in ascitic cancer cells (Fig. 1G, H). Consistent with their disseminating capacity, ascitic cancer cells selectively up-regulated genes associated with cell migration, CXCR chemokine receptor binding, cytokine–cytokine receptor interaction, and chemokine signaling. Gene set enrichment analysis (GSEA) also confirmed the results of GO term and KEGG analysis (Fig. 1J, K), which were further independently validated by qRT-PCR (Fig. S1). Taken together, peritoneal tissues, the preferential OC metastasis site, reactivated and stimulated the proliferation of the disseminating OC cells that were forced into quiescence of low metabolic activity by malignant ascites.

### Long-range modulation of OC cell proliferation and migration by ADSCs

**Figure 2:**
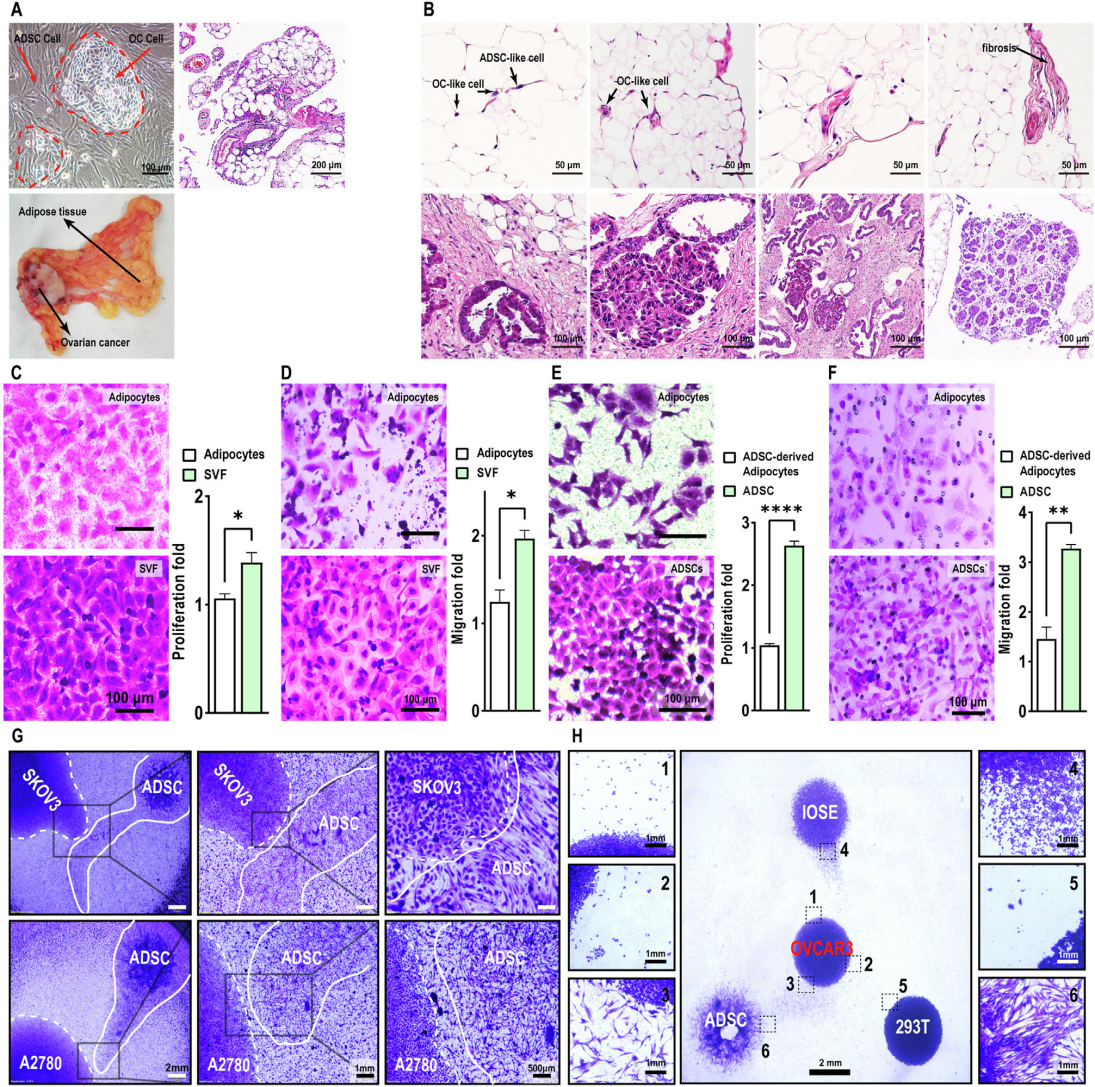
Long-range regulation of OC cell proliferation and migration by ADSCs. **(A)** OC cells formed islands surrounded by adipose tissue *in vivo* and surrounded by ADSCs *in vitro*. Scale bar = 100 or 200 μm. **(B)** Hematoxylin & eosin staining of peritoneal OC samples showed the interaction between OC and adipose tissue. Scale bar = 50 or 100 μm. **(C, D)** The ADSCs-rich SVF stimulated OC cell migration and proliferation more than primary adipocytes. Scale bar = 100 μm. **(E, F)** ADSCs stimulated OC cell migration and proliferation more than their differentiated adipocyte progenies. Scale bar = 100 μm. **(G)** A mutual migratory stream formed between ADSCs and OC cells seeded as islands at a distance. Scale bar = 2 mm, 1 mm, or 500 μm. **(H)** OC cells readily formed a migratory stream with ADSCs but not with IOSE80 or 293T cells. Scale bar = 2 or 1 mm. All data represent mean ± standard error of the mean (^∗^*P* < 0.05, ^∗∗^*P* < 0.01, ^∗∗∗∗^*P* < 0.0001). OC, ovarian cancer; SVF, stromal vascular fraction; ADSCs, adipose-derived stem cells.

The omentum, the most common metastatic site for OC, is essentially a fat pad, most enriched in adipose tissues (Fig. 2A). When digested and *in vitro* cultured, omental adipose and ovarian cancer tissue, instead of forming a mixed culture of heterogeneous cancer and adipose-derived cells, reacquired their *in vivo* spatial arrangement in which primary OC cells formed central “islands” surrounded by omental adipose cells (Fig. 2A). Hematoxylin and eosin staining of peritoneal OC samples showed that OC cells could recruit ADSC-like cells and induce fibrosis. As OC cells continuously induced ADSC-like cells to undergo fibrosis and consumed lipids from adipocytes, OC progressed and gradually occupied the peritoneal cavity (Fig. 2B). To discern the role of adipocytes and/or ADSCs in OC progression and metastasis, we isolated patient peritoneal adipocytes and stromal vascular fraction containing ADSCs and subsequently cocultured them with SKOV3 cells. Although both adipocytes and stromal vascular fraction stimulated the migration and proliferation of SKOV3 cells (Fig. 2C, D), the stromal vascular fraction was significantly more potent than primary adipocytes. In addition, ADSCs promoted more migration and proliferation than their differentiated adipocyte progenies (Fig. 2E, F).

Next, to examine possible communication between patient-derived ADSCs and OC cells, we opted to seed them as isolated spots separated by distance. After a 24-h incubation, mutual migration was evident between the isolated spots, strongly suggesting cell–cell communication through certain secretory mechanisms (Fig. 2G). Further examination revealed that ADSCs actively migrated towards OC cells, somewhat resembling a homing mechanism that might occur during metastasis *in vivo*.

### OC cells preferentially migrate towards ADSCs

Diverse tissues are present in the peritoneal cavity, but OC cells have a strong predilection for metastasizing to peritoneal adipose tissues. According to the above results, we sought to develop a competitive cell migration assay and examine whether ADSCs could have a stronger interaction with OC cells than with other relevant cells in the abdomen. For their relevance, we incorporated IOSE80 (human ovarian surface epithelial cells) and 293T (human kidney) cells in the competition assays. We seeded cells as islands at equal distances from OC cells and monitored their migratory behaviors over time. OC cells readily formed a migratory stream with ADSCs but not with IOSE80 or 293T cells (Fig. 2H). This result is reminiscent of observations *in vivo*, suggesting a potential role of ADSCs in OC metastasis to omental sites.

### ADSCs-EVs mediate the effects of ADSCs on OC cell proliferation and migration

**Figure 3:**
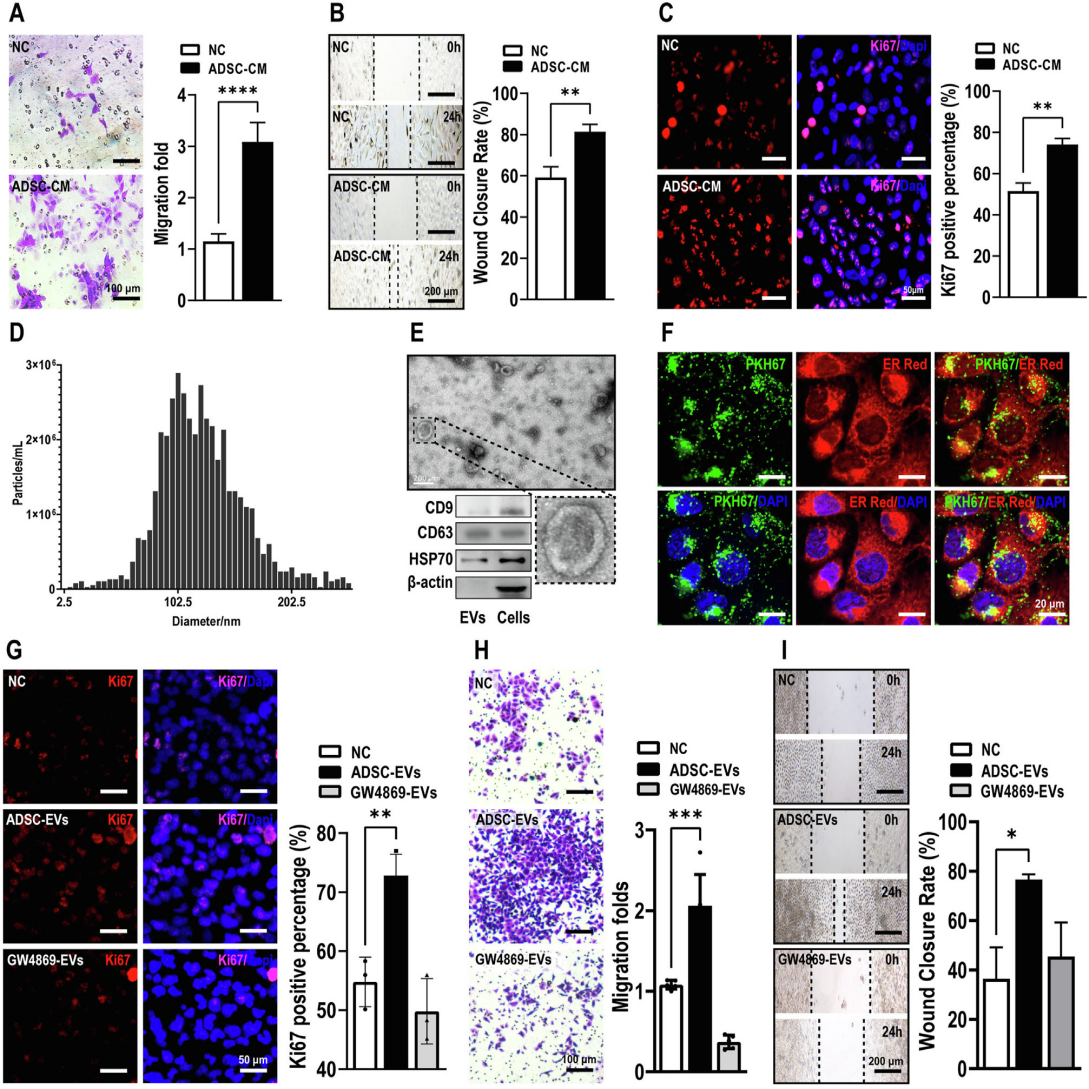
EVs mediate the effects of ADSCs on OC cell proliferation and migration. **(A**–**C)** ADSCs-CM stimulated OC cell migration, accelerated wound closure, and enhanced OC cell proliferation. Scale bar = 100, 200, or 50 μm. **(D, E)** Confirmation and characterization of ADSCs-EVs by nanoparticle tracking analysis, transmission electron microscopy, and western blot analysis. Scale bar = 200 nm. **(F)** Uptake of PKH67-labeled EVs by OC cells. Scale bar = 20 μm. **(G)** ADSCs-EVs stimulated OC cell proliferation, and this effect was abolished by the EV biogenesis inhibitor GW4869. Scale bar = 50 μm. **(H, I)** ADSCs-EVs stimulated OC cell wound closure and migration, and these effects were abolished by the EV biogenesis inhibitor GW4869. Scale bar = 100 or 50 μm. All data represent mean ± standard error of the mean (^∗^*P* < 0.05, ^∗∗^*P* < 0.01, ^∗∗∗^*P* < 0.001, ^∗∗∗∗^*P* < 0.0001). OC, ovarian cancer; ADSCs, adipose-derived stem cells; ADSCs-CM, ADSCs-conditioned medium; EV, extracellular vesicle; ADSCs-EVs, ADSCs-derived extracellular vesicles.

Our results suggested that a secretory mechanism might underlie the interaction between ADSCs and OC cells. In strong support of this speculation, ADSCs-CM strongly stimulated 10.13039/100003224OC cell migration and accelerated wound closure (Fig. 3A, B). ADSCs-CM also significantly increased OC cell proliferation, as determined by Ki67 staining (Fig. 3C).

EVs are nanovesicles increasingly recognized as a major vehicle between cells at different organ sites that are often hijacked by cancer cells for their perpetuation.^14^^,^^22^ Consequently, we isolated and confirmed the characteristics of EVs from ADSCs-CM (Fig. 3D, E). When added to the medium, PKH67-labeled ADSCs-EVs efficiently entered OC cells. Within 10 min, strong PKH67 signals were detected inside OC cells and were able to persist for at least a day (Fig. 3F). Phenotypically, ADSCs-EVs treatment nearly doubled the number of proliferating (Ki67^+^) cells (Fig. 3G) and dramatically stimulated OC cell wound healing and migration (Fig. 3H, I). Conversely, the inhibition of ADSCs-EVs production using the small molecule inhibitor GW4869 effectively abolished the stimulatory effects of ADSCs on OC cell proliferation, wound healing, and migration (Fig. 3G–I). Taken together, these data strongly suggest that EVs may underlie the interaction between ADSCs and OC cells.

### Transcriptome analysis identified major pathways and biological processes altered by ADSCs-EVs treatment

**Figure 4:**
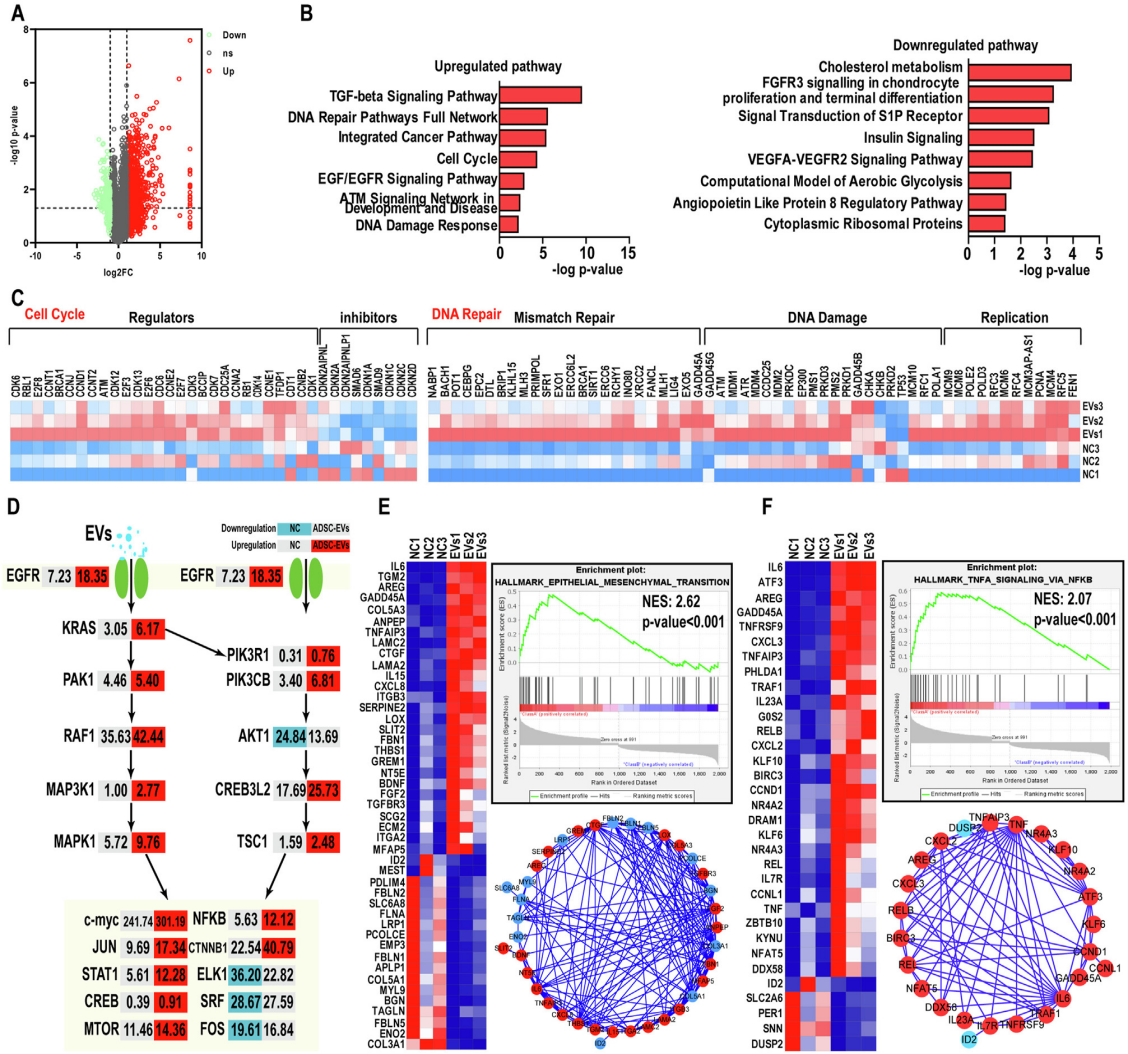
Transcriptome analysis identifies major pathways and biological processes related to tumor progression and metastasis altered by ADSCs-EVs treatment. **(A)** Differentially expressed genes (DEGs) with a fold change cut-off of 2-fold between control and ADSCs-EVs-treated OC cells. **(B)** The up-regulated DEGs were predominantly enriched in TGF-beta, DNA repair, cell cycle, and EGF/EGFR signaling; cholesterol metabolism, FGFR3 signaling, S1P receptor, and insulin signaling were modestly down-regulated. **(C)** Numerous genes related to the cell cycle and DNA replication, damage, and repair were altered. **(D)** Extensive alterations in major EGF/EGFR signaling genes were identified by KEGG pathway analysis. **(E, F)** Epithelial-mesenchymal transition and the NF-κB signaling pathway, two key processes involved in tumor progression and metastasis, were ranked first and second, respectively, in GSEA. OC, ovarian cancer; ADSCs, adipose-derived stem cells; ADSCs-EVs, ADSCs-derived extracellular vesicles; EGFR, epidermal growth factor receptor; EGF, epidermal growth factor; NF-κB, nuclear factor kappa B.

To investigate the underlying mechanism, we performed transcriptome analysis of OC cells with and without ADSCs-EVs treatment and obtained a global landscape of altered genes and biological processes. In ADSCs-EVs-treated OC cells, 3082 genes were up-regulated and 312 were down-regulated 2-fold (Fig. 4A). The KEGG analysis revealed that several crucial biological processes, including the cell cycle and DNA replication, damage, and repair were significantly up-regulated by ADSCs-EVs (Fig. 4B, C). Among them, cell cycle inhibitors, including Smads and CDKNs, were down-regulated while CDKs, CCNs, E2Fs ATM, ATR, ERCC6, MCMs, and BRCA1 were up-regulated (Fig. 4C), consistent with a tumor-promoting effect (Fig. 4C).

Importantly, several pathways, such as the TGF-β and EGF/EGFR signaling pathways, which are closely associated with cancer progression and metastasis, were found to be highly enriched with ADSCs-EVs treatment (Fig. 4B–D). Among the up-regulated genes were several key EGFR pathway genes, including EGFR, Ras, and MAP3K1 (Fig. 4D). GSEA further confirmed that ADSCs-EVs may promote OC progression and metastasis by facilitating epithelial-to-mesenchymal transition and up-regulating the NF-κB pathway (Fig. 4E, F). Numerous crucial genes involved in these two processes were significantly altered. For example, the gene expression of IL-6, TGM2, and CXCL8, among others, was up-regulated more than 5-fold, while the gene expression of FBLN2, PCOLCE, and FBLN1 was down-regulated over 2-fold (Fig. 4E, F).

### EGF drivers the ADSCs-EVs-mediated proliferation and migration of OC cells

**Figure 5:**
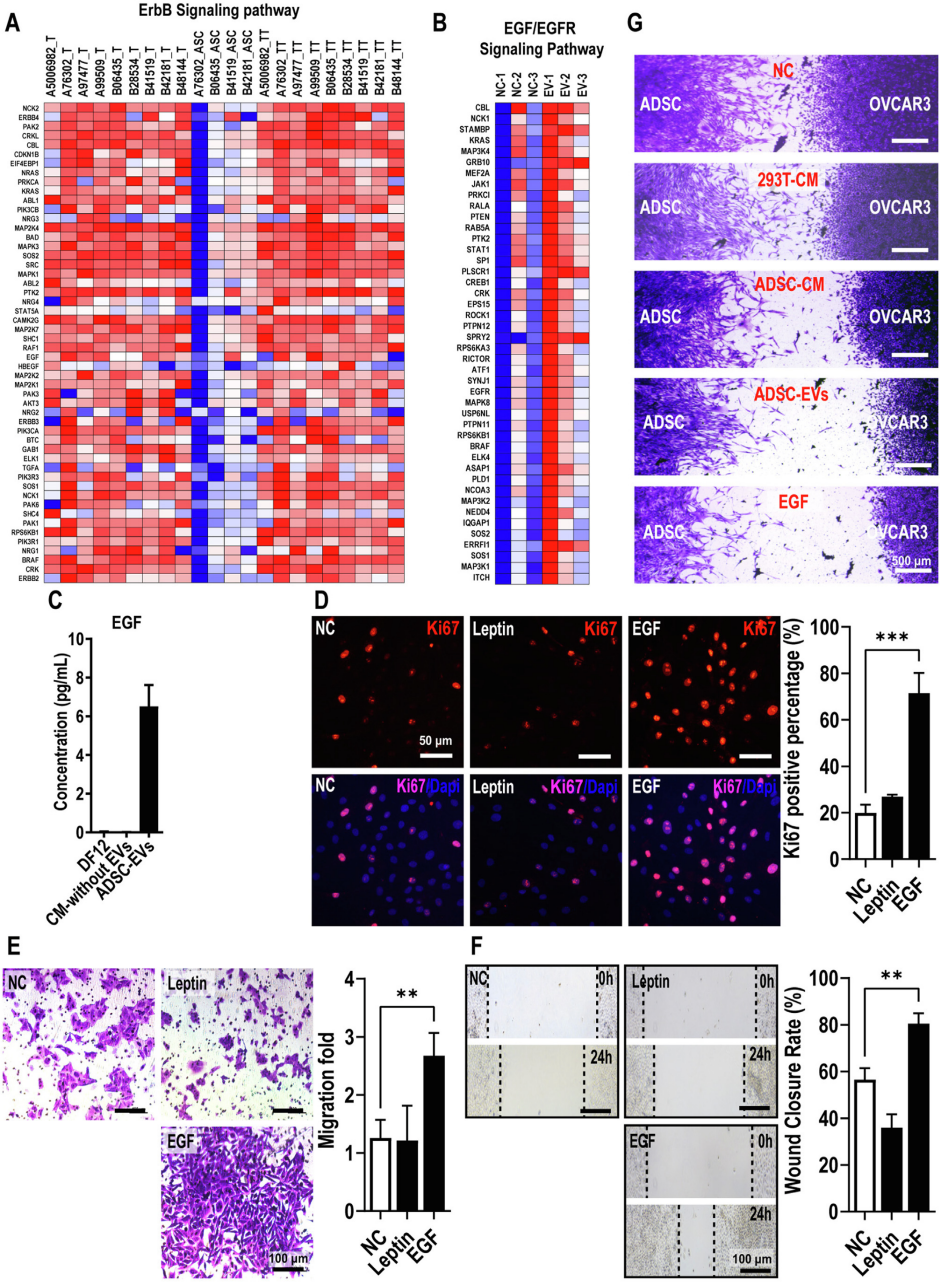
ADSCs-EVs contain and deliver EGF to OC cells. **(A, B)** The heatmap revealed that the EGFR signaling pathway was significantly up-regulated during peritoneal OC metastasis and/or upon ADSCs-EVs treatment. **(C)** The presence of EGF in ADSCs-EVs but not EVs-depleted ADSCs-CM was determined by enzyme-linked immunosorbent assay. **(D**–**F)** EGF but not adipocyte-secreted leptin stimulated OC cell proliferation and accelerated OC cell migration and wound closure. Scale bar = 50 or 100 μm. **(G)** The addition of ADSCs-CM, ADSCs-EVs, and EGF but not 293T-CM effectively abolished the mutual migration between ADSCs and OC cells. Scale bar = 500 μm. All data represent mean ± standard error of the mean (^∗∗^*P* < 0.01, ^∗∗∗^*P* < 0.001). OC, ovarian cancer; ADSCs, adipose-derived stem cells; ADSCs-EVs, ADSCs-derived extracellular vesicles; EGFR, epidermal growth factor receptor; EGF, epidermal growth factor; ADSCs-CM, ADSCs-conditioned medium.

ADSCs secrete abundant EGF, the most common carcinogenic molecule, and are capable of activating diverse downstream signaling pathways. Dysregulation of the EGF/EGFR signaling pathway has been shown to contribute to tumor initiation and progression.^36^ Our transcriptome analysis revealed a significant up-regulation of the EGF/EGFR signaling pathway during peritoneal metastasis (Fig. 5A) and with ADSCs-EVs treatment (Fig. 5B). Then, we utilized enzyme-linked immunosorbent assay to investigate the presence of EGF in ADSCs-CM and/or EVs, and EGF was mostly detected in ADSCs-EVs, strongly suggesting that EGF is primarily present in EVs (Fig. 5C). When EGF was added to the basal culture medium, it mimicked the activity of ADSCs-CM, enhancing OC cell proliferation, migration, and wound healing capabilities (Fig. 5D–F). In contrast, leptin, a major adipokine secreted by adipocytes and commonly used as a control, did not produce a similar effect. Instead, leptin could hinder the proliferation and migration of OC cells (Fig. 5D–F).

To further confirm that EGF was a main factor underlying ADSCs-EVs-mediated effects, we developed a second competitive cell migration assay. As with the protocol illustrated in Figure 2, we seeded OC cells and ADSCs as islands at a distance and incubated them in the indicated media. As previously observed, mutual migration was apparent between OC cells and ADSCs in a fresh medium (Fig. 5G). The negative control 293T-CM did not cause a detectable impact on this mutual migration. In contrast, both ADSCs-CM and ADSCs-EVs abolished this mutual migration (Fig. 5G). The blocking effect of EGF was similar to that observed with ADSCs-CM and ADSCs-EVs (Fig. 5G). Taken together, our results demonstrate that EGF underlies the function of ADSCs-EVs.

### ADSCs-EVs treatment enhanced EGFR signaling

**Figure 6:**
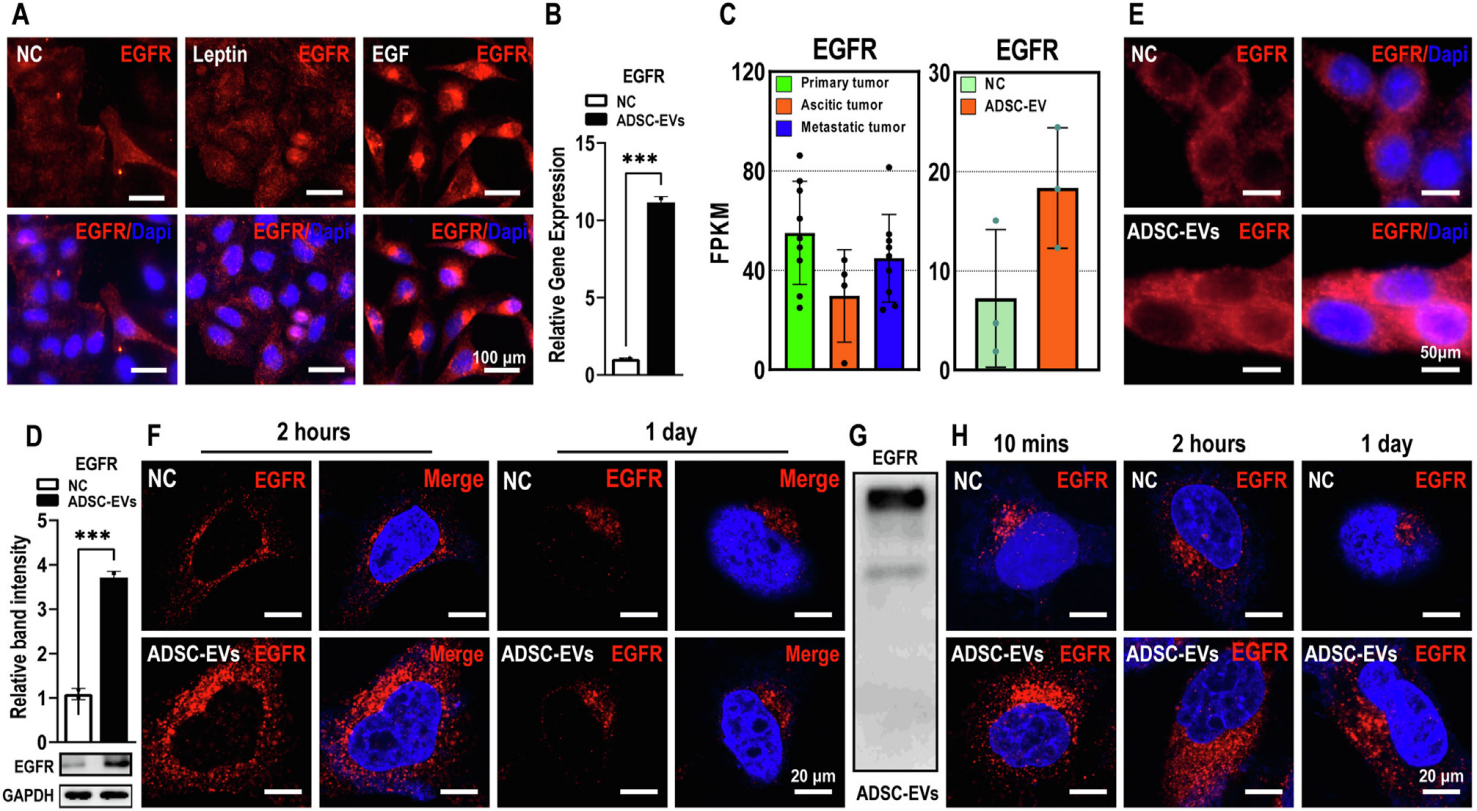
ADSCs-EVs contain and deliver EGFR to OC cells. Scale bar = 100 μm. **(A)** EGFR expression was up-regulated by EGF but not by leptin. **(B**–**E)** EGFR was up-regulated by ADSCs-EVs treatment, as determined by quantitative reverse transcription PCR (B), RNA-sequencing FPKM data (C), western blot analysis (D), and immunofluorescence staining (E). Scale bar = 50 μm. **(F)** EGFR was up-regulated at the 2-h time point but returned to the control level overnight. Scale bar = 20 μm. **(G)** Western blot analysis proved the presence of EGFR in ADSCs-EVs. Scale bar = 20 μm. **(H)** Closer examination revealed that ADSCs-EVs treatment resulted in up-regulation of EGFR as early as 10 min and that the up-regulation decreased over time. Scale bar = 20 μm. All data represent mean ± standard error of the mean (^∗∗∗^*P* < 0.001). OC, ovarian cancer; ADSCs, adipose-derived stem cells; ADSCs-EVs, ADSCs-derived extracellular vesicles; EGFR, epidermal growth factor receptor; EGF, epidermal growth factor.

Next, we examined EGFR expression by immunofluorescence staining and found that EGF stimulated the expression of EGFR in OC cells (Fig. 6A). In contrast, leptin did not have any detectable effect. Moreover, the expression of EGFR in OC cells was up-regulated over 10-fold at the mRNA level and 3-fold at the protein level by ADSCs-EVs (Fig. 6B–E).

Interestingly, we found that EGFR staining in ADSCs-EVs-treated OC cells was already stronger than that in control cells at the 2-h time point, but decreased overnight (Fig. 6F). Since it is well documented that cancer cell-derived EVs deliver EGFR to other cells,^37^ we sought to determine whether ADSCs-EVs could also contain and deliver EGFR to OC cells. We performed western blot analysis and confirmed the presence of abundant EGFR protein in ADSCs-EVs (Fig. 6G). These ADSCs-EVs delivered EGFR proteins readily into OC cells (Fig. 6H). At 10 min, the earliest time point examined, EGFR staining in ADSCs-EVs-treated OC cells was already much stronger than that in control cells. The intensity of EGFR staining appeared to decrease progressively throughout the experiment (Fig. 6H), suggesting that the increase in EGFR was mainly from ADSCs-EVs. Taken together, our results demonstrate that the EGFR signaling pathway underlies ADSCs-mediated effects on OC cell proliferation and migration at multiple levels.

### ADSCs-EVs treatment stimulated NF-κB signaling and inflammatory pathways

**Figure 7:**
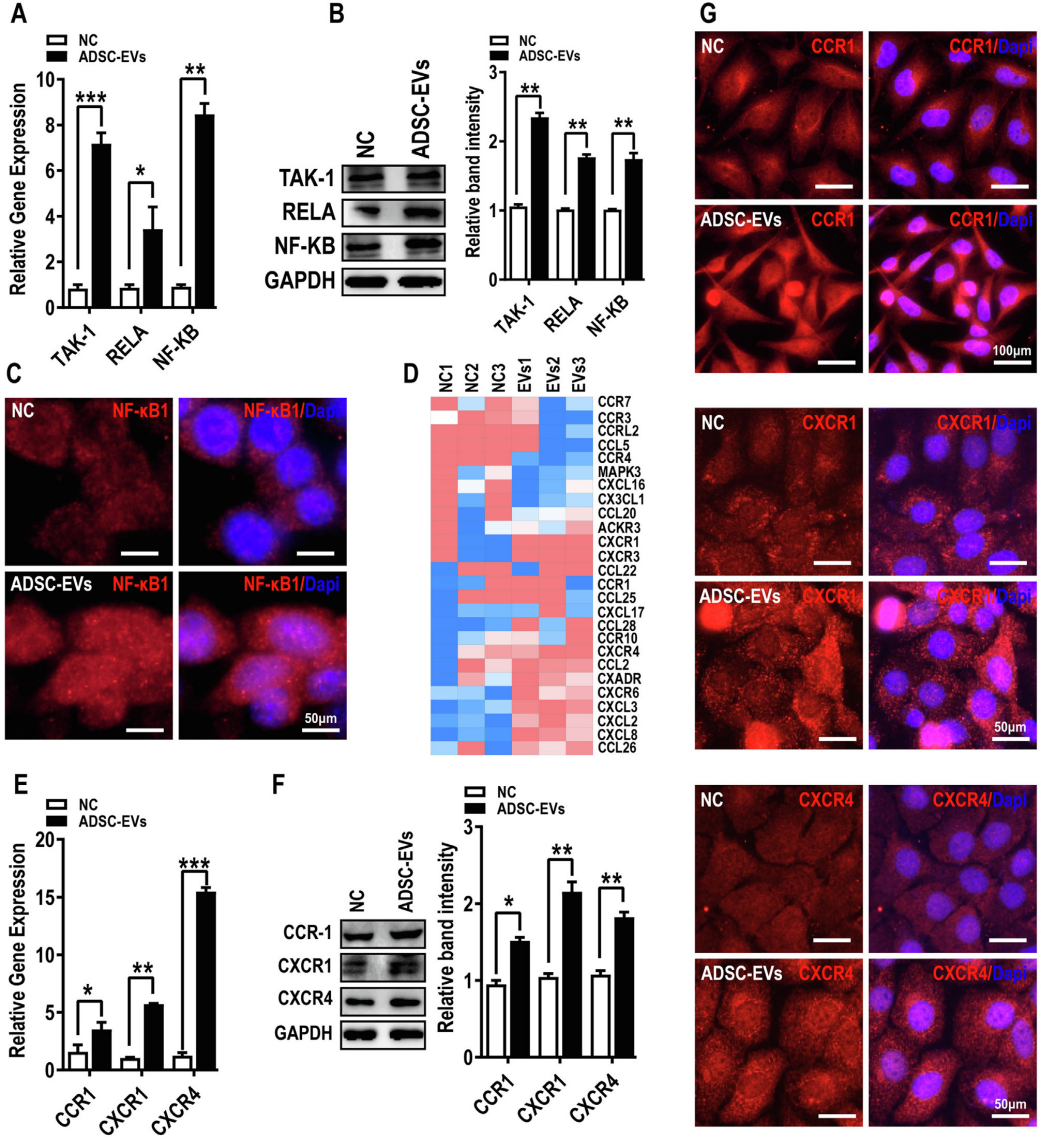
ADSCs-EVs treatment stimulates NF-κB signaling and inflammatory pathways. **(A**–**C)** The NF-κB pathway was up-regulated in ADSCs-EVs-treated OC cells, as determined by quantitative reverse transcription PCR (A), western blot analysis (B), and immunofluorescence staining (C). Scale bar = 50 μm. **(D)** Numerous genes associated with chemotaxis pathways, in particular chemokine, cytokine, and receptor genes, were altered by ADSCs-EVs treatment. **(E**–**G)** ADSCs-EVs treatment up-regulated the expression of chemotaxis-related genes, as determined by quantitative reverse transcription PCR (E), western blot analysis (F), and immunofluorescence staining (G). Scale bar = 100 or 50 μm. All data represent mean ± standard error of the mean (^∗^*P* < 0.05, ^∗∗^*P* < 0.01, ^∗∗∗^*P* < 0.001). OC, ovarian cancer; ADSCs, adipose-derived stem cells; ADSCs-EVs, ADSCs-derived extracellular vesicles; NF-κB, nuclear factor kappa B.

The NF-κB signaling pathway, which is frequently involved in tumor progression and metastasis, was the third most enriched pathway in our GSEA and one of the top four pathways in our KEGG pathway analysis. Consistent with the transcriptome analysis, ADSCs-EVs treatment significantly boosted the expression of vital NF-κB signaling molecules, including NF-κB, RELA, and TAK1, as assessed by qRT-PCR, western blot analysis (Fig. 7A, B), and immunofluorescence staining (Fig. 7C).

NF-κB is a master regulator of inflammation that plays a critical role in cancer progression and metastasis.^38^ Similar to NF-κB signaling, a range of inflammatory pathways were induced upon ADSCs-EVs treatment, including many potential NF-κB signaling inflammatory targets, such as CCRs and CXCLs (Fig. 7D, E). Among them, we selected CCR1, CXCR1, and CXCR4 and confirmed their up-regulation by qRT-PCR, western blot analysis, and immunostaining (Fig. 7F, G).

### The EGFR-NF-κB axis underlies ADSCs-EVs-mediated effects on OC cells

**Figure 8:**
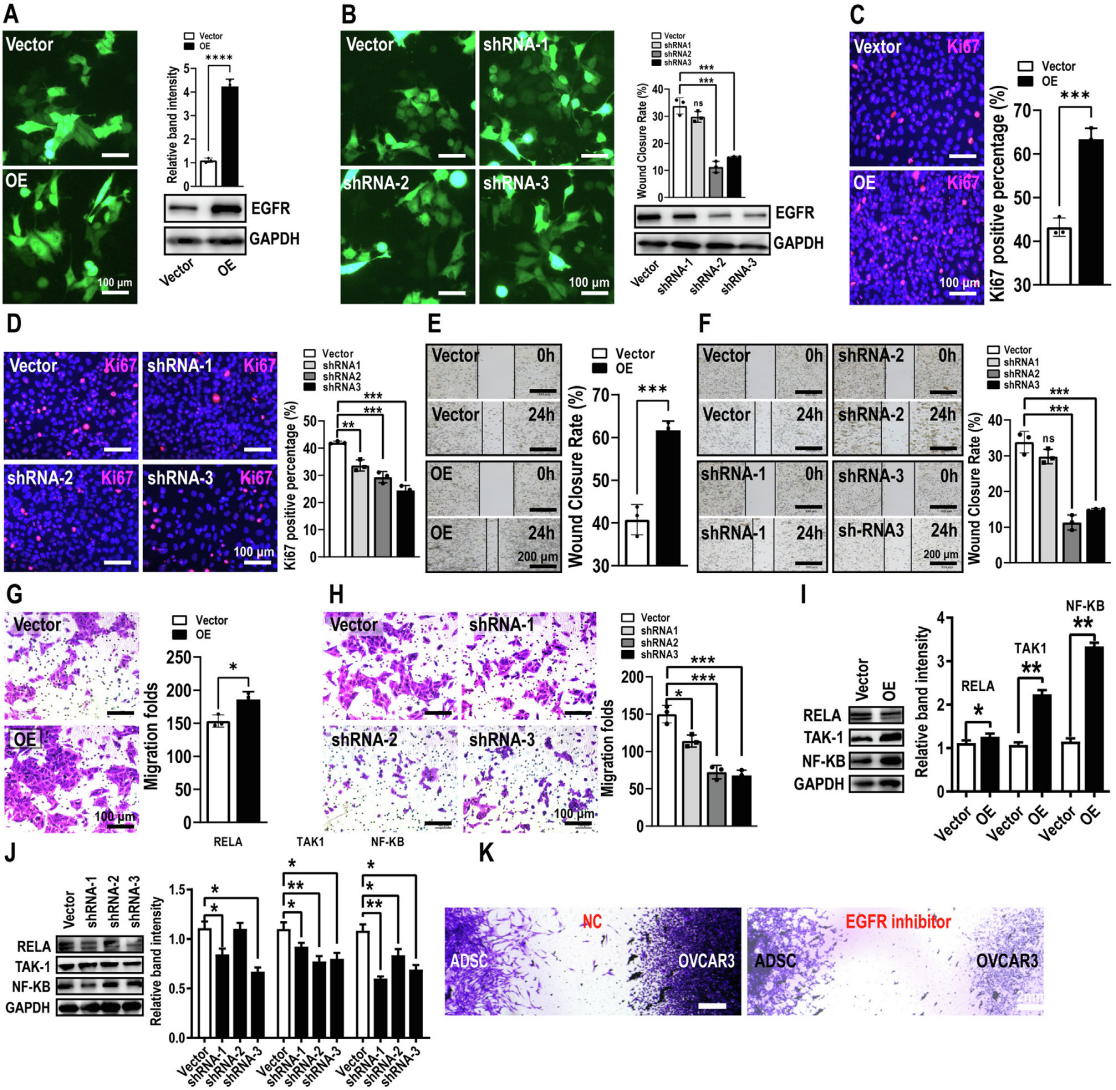
The EGFR and NF-κB signaling pathways underlie ADSCs-EVs-mediated effects on OC cells. **(A, B)** Confirmation of successful EGFR overexpression (A) and shRNA-mediated knockdown (B) by western blot analysis. Scale bar = 100 μm. **(C, D)** EGFR overexpression increased (C) and shRNA knockdown decreased (D) OC cell proliferation, as determined by Ki67 staining. Scale bar = 100 μm. **(E**–**H)** EGFR overexpression enhanced (E, G) and shRNA knockdown attenuated (F, H) OC cell migration, as determined by wound healing and transwell assays. Scale bar = 200 or 100 μm. **(I, J)** NF-κB signaling molecules were up-regulated by EGFR overexpression and down-regulated by shRNA knockdown. **(K)** EGFR inhibitors effectively abolished the ADSCs-EVs-mediated increase in OC cell migration. Scale bar = 2 mm. All data represent mean ± standard error of the mean (^∗^*P* < 0.05, ^∗∗^*P* < 0.01, ^∗∗∗^*P* < 0.001, ^∗∗∗∗^*P* < 0.0001). OC, ovarian cancer; ADSCs, adipose-derived stem cells; ADSCs-EVs, ADSCs-derived extracellular vesicles; EGFR, epidermal growth factor receptor; NF-κB, nuclear factor kappa B.

Subsequently, we aimed to ascertain whether the EGFR and NF-κB pathways could form a functionally important axis. To this end, we generated EGFR overexpression and knockdown constructs and confirmed successful EGFR overexpression and knockdown in OC cells by western blot analysis (Fig. 8A, B). As expected, EGFR overexpression increased and EGFR knockdown inhibited OC cell proliferation (Fig. 8C, D) and migration (Fig. 8E–H). Importantly, at the molecular level, the overexpression of EGFR increased while the knockdown of EGFR suppressed NF-κB pathway-related gene expression (Fig. 8I, J). Finally, we performed competitive inhibition assays and found that the EGFR inhibitor canertinib effectively abolished the mutual migration between ADSCs and OC cells (Fig. 8K). Taken together, these results demonstrate that the EGFR-NF-κB axis is important for ADSCs-EVs-mediated effects on OC cell proliferation and migration.

## Discussion

Communication and interaction between cancer and stromal cells are integral parts of the tumor microenvironment that promote the growth, metastasis, and drug resistance of cancer cells. Peritoneal dissemination and malignant ascites are characteristics of advanced OC.^11^ However, limited data are available regarding the specific stromal cell types and mechanisms that contribute to OC progression and metastatic tropism for peritoneal adipose tissue. The present study suggests that peritoneal ADSCs participate in OC progression via EVs-mediated long-range communication. Through ADSCs-EVs-mediated delivery of key signaling molecules (*e.g.*, EGF and EGFR) to OC cells, ADSCs reactivate the EGFR-NF-κB axis and promote peritoneal OC metastasis.

The stromal cells found in peritoneal adipose tissue typically consist of adipocytes,^39^ tumor-infiltrating inflammatory cells,^40^ vascular endothelial cells,^41^ and ADSCs.^22^ Among other cells, adipocytes are believed to assume a central role in the development of the omental metabolic microenvironment that favors OC metastasis and colonization.^39^ Cancer cells can attract and reprogram adipocytes into “cancer-associated adipocytes” that facilitate aggressive growth and OC metastasis.^42^ However, it remains to be determined whether adipocytes but not ADSCs contribute to this process. In the present study, we isolated adipocytes and ADSCs from OC patients. Our results showed that ADSCs enhanced OC proliferation and migration more than adipocytes. Notably, EGF, a growth factor secreted by ADSCs, exhibited a significantly stronger effect on the proliferation and migration of OC cells than leptin, a major cytokine secreted by adipocytes. Our findings are consistent with an alternative mechanism suggesting that ADSCs are a major mediator of omental OC metastasis. Similar to our findings, the potential involvement of ADSCs in OC progression was suggested early by several other groups.^7^^,^^43^^,^^44^ However, a strong association between OC progression and ADSCs *in vivo* remains to be established.^45^^,^^46^

Our study also extends earlier reports by showing that peritoneal ADSCs mediate long-range modulation of OC progression and metastasis via EVs. EVs are nanovesicles loaded with microRNAs, mRNAs, and proteins from the cells of origin and serve as a major long-range communication pathway between the cells of origin and distant target cells/tissues.^47^ Through binding with tissue- and/or cell-specific membrane receptors or ligands, EVs can deliver specific signals to target cells and tissues. During tumor progression, cancer cells can secrete and hijack EVs for perpetuation. Gastric cancer cell-derived EVs can deliver EGFR to remodel the liver microenvironment and promote liver-specific metastasis.^37^ EGFR-containing EVs from lung cancer cells have been shown to stimulate VEGF production in endothelial cells and induce tumor-specific regulatory T cells.^48^

In contrast to the aforementioned studies, the results obtained in the current study suggest that peritoneal ADSCs can utilize EVs to deliver EGFR signaling molecules to reprogram the proliferative and migratory properties of tumor cells, promoting OC progression and metastasis. We developed long-range migration and competitive inhibition assays and showed the functional importance of ADSCs-EVs-mediated long-range modulation of OC cells. Notably, peritoneal ADSCs regulate OC progression and metastasis through multiple mechanisms. ADSCs-EVs not only transport EGF but also deliver EGFR to OC cells. We hypothesize that EGFR signaling may be the central node mediating this interaction between peritoneal ADSCs and OC cells.

Downstream of EGFR signaling, diverse pathways (*e.g.*, epithelial-mesenchymal transition and p53 signaling) were altered. In particular, the NF-κB pathway, a focal regulatory point in inflammation and the immune response was dramatically up-regulated by ADSCs-EVs and likely at least partially accounted for cancer progression and metastasis.^49^

Clinically, it is particularly intriguing that peritoneal ADSCs may remotely modulate OC cells through ADSCs-EVs-mediated delivery of multiple EGFR signaling molecules, as observed in our study. EGFR signaling, which is dysregulated in more than 70% of OC patients, is associated with high tumor mortality.^50^ EGFR-targeted therapies are widely used for breast,^51^ colorectal,^52^ and non-small cell lung cancers.^53^ However, EGFR inhibitors have not shown promising therapeutic effects in OC, and the mechanism underlying resistance to EGFR inhibitors remains unclear. Our findings may provide novel guidance for further research to overcome this resistance. Since peritoneal ADSCs supply EGFR signaling molecules to OC cells via ADSCs-EVs, EGFR-targeted therapy combined with adjuvant agents that specifically block the paracrine effect of ADSCs and/or target ADSCs-EVs could be a viable approach. Given the complex nature and the high mortality rate associated with advanced OC, further *in vivo* explorations are warranted to determine the roles of peritoneal ADSCs-EVs in OC progression, metastasis, and drug resistance.

In conclusion, we discovered that stem cells and stem cell-derived EVs in adipose tissue play an important role in reactivating quiescent ovarian cancer cells from ascites. Our study also shows that peritoneal ADSCs-EVs-mediated delivery of EGFR signaling molecules (*e.g.*, EGF and EGFR) modulates OC cell proliferation and migration at multiple levels, likely a key mechanism promoting OC progression and peritoneal dissemination.

## Ethics approval

This study was approved by The Ethics Committee of Shanghai Tenth People's Hospital (No. SHDSYY-2021-4446; Title: Role and mechanism of adipose derived stem cells in promotion of proliferation and metastasis of ovarian cancer cells. Date of approval: March 22, 2021). The tissue samples were obtained with written informed consent from each patient.

## Author contributions

**Lian Wang**: conceptualization, methodology, formal analysis, investigation, writing-original, funding acquisition; **Ning Luo**: validation, data curation, visualization, writing-original, and conceptualization; **Jihui Zhu**, **Wenhan Yang**, **Guihai Ai**, **Shengkai Jin**, and **Xue Zhang**: validation, data curation, and visualization; **Weihong Yang**: validation, data curation, visualization, and investigation; **Ke Hu**: validation; **Yantao Fan**: investigation and resources; **Xiaowen Shao**: data curation, investigation, and resources; **Dan Deng**: supervision and project administration; **Zhongping Cheng**: supervision, project administration, conceptualization, and funding acquisition; **Zhengliang Gao**: supervision, project administration, conceptualization, funding acquisition, and writing-review & editing.

## Conflict of interests

The authors declared no conflict of interests.

## Funding

This work was supported by funds from the National Key R&D Program of China (No. 2019YFA0110300 to ZLG) and the National Natural Science Foundation of China (No. 31900522 to LW, 81773302 to YTF, 32370895, 82373269 to ZPC, 32070862 to ZLG). It was also supported by the Fundamental Research Funds for the Central Universities of China (project plan 22120210588), the Climbing Talent Program of Shanghai Tenth People's Hospital (China) (No. 2021SYPDRC036), and the China Postdoctoral Science Foundation (No. 2019M651571).

## Data availability

The data supporting the findings of this study are available from the corresponding author upon reasonable request.
